# An endoparasitoid wasp influences host DNA methylation

**DOI:** 10.1038/srep43287

**Published:** 2017-02-23

**Authors:** Sunil Kumar, Yonggyun Kim

**Affiliations:** 1 Department of Bioresource Sciences, Andong National University, Andong 36729, Korea

## Abstract

Parasitism by endoparasitoid wasps changes the expression of various host genes, and alters host immune and developmental processes. However, it is not clearly understood how parasitism changes host gene expression in a whole genome scale. This study focused on an epigenetic control of *Cotesia plutellae*, an endoparasitoid wasp, against its host, *Plutella xylostella*. Two DNA methyltransferases (*DNMT-1* and *DNMT-2*) are encoded in the genome of *P. xylostella*. In addition, *methyl-binding domain* proteins (*MBD*s) and DNA demethylation factor, *ten-eleven translation* protein (*TET*) are encoded. DNA methylation of *P. xylostella* genomic DNA was confirmed by restriction digestion with *Gla* I specific to 5-methylcytosine. DNA methylation intensity in parasitized (P) larvae was decreased compared to that in nonparasitized (NP) larvae, especially at late parasitic stage, at which expression levels of both *DNMT-1* and *DNMT-2* were also decreased. DNA demethylation of *P. xylostella* was confirmed in both NP and P larvae by restriction digestion with *PvuRts*1I recognizing 5-hydroxymethyl cytosine. Parasitism also suppressed expression levels of *TET* and *MBD*s. Treatment of 5-aza-2′-deoxycytidine (AZA) reduced DNA methylation intensity of NP larvae, causing suppression of hemocyte-spreading behavior and delay of immature development. RNA interference of *DNMT-1* or *DNMT-2* mimicked the adverse effects of AZA.

Parasitism is a non-mutualistic symbiosis. Its success requires host regulation that is beneficial to parasites. Endoparasitoids including some braconid and ichneumonid wasps exhibit koinobiotic life, in which wasp larvae grow inside developing hosts^1^. These wasps can regulate hosts through significant immunosuppression to defend the attack of the host’s immune system and induce the delay of host development to allow endoparasitoid larvae to mature first^2^. To achieve various ranges of host regulation, massive changes in host gene expression levels occur as seen in different parasitic patterns induced by braconid or ichneumonid wasps^3^,^4^. Indeed, some parasitic factors derived from polydnaviruses symbiotic to endoparasitoids are transcriptional regulators, such as viral ankyrins (vankyrins) and viral histone H4 (vH4). Vankyrins are truncated variants of host inhibitor κB that plays a crucial role in inhibiting the activity of nuclear factor κB, a transcriptional factor^5^,^6^. Some vankyrins have been demonstrated to be able to inhibit host gene expression^7^. A vH4 has been identified in Cotesia plutellae bracovirus (CpBV). It has an extended N-terminal tail (38 amino acids containing 9 lysine residues) compared to host histone H4^8^. vH4 joins host nucleosomes and alters host gene expression^9^. Thus, some parasitic factors can modulate host gene expression. However, it was not clearly understood how the massive changes in host gene expression occurred during parasitism.

Epigenetics deals with heritable changes in gene expression without change in DNA sequence. DNA methylation, histone modification, and microRNA expression are examples of epigenetic control of gene expression^10^. Environmental changes such as nutrients^11^, exposure to pesticides^12^, pathogens^13^, and climate change^14^ can influence epigenetic control. Especially, DNA methylation on a specific genome in response to environmental change is heritable by its *de novo* maintenance devices^15^. DNA methyltransferases (DNMTs) are classified into three types (DNMT-1, DNMT-2, and DNMT-3), in which DNMT-2 is no longer considered as a DNA methylation agent due to its specificity to tRNA in vertebrates^16^,^17^. *De novo* DNA methylation is performed by DNMT-3, while the methylation state is maintained by catalytic activity of DNMT-1. In insects, DNMT-1 and DNMT-2 are well conserved whereas DNMT-3 is uncommon except some hymenopteran and hemipteran insects^18^. As reported in *Drosophila*, insect DNA methylation levels are much lower compared to those of vertebrates^19^. Unlike vertebrate genomic DNAs that are globally methylated, insect genomes are locally methylated in gene body probably to regulate the transcription and mRNA splicing of target genes^20^.

Endoparasitoid wasp *C. plutellae* parasitizes young larvae of the diamondback moth (DBM), *Plutella xylostella*^21^. Parasitized larvae exhibit significant alterations in immune response and development^22^. A proteomic analysis has indicated that more than 25% of host genes annotated into various categories of physiological functions are altered in gene expression after being parasitized^3^. In this study, we hypothesized that *C. plutellae* parasitism could alter host gene expression in an epigenetic mode by changing DNA methylation level in addition to manipulating the activities of host transcriptional factors. To test this hypothesis, DNA methylation was monitored in *P. xylostella* and the DNA methylation levels in parasitized (P) larvae were compared to those in nonparasitized (NP) larvae. Based on the presence of DNA methylation, DNA methylation/demethylation-associated genes were identified from *P. xylostella* genome and their expressions in both NP and P larvae were assessed. Finally, this study demonstrated the effect of down-regulation of DNA methylation on immune response and immature development of *P. xylostella*.

## Results

### DNA methylation on *P. xylostella* genome

**Figure 1:**
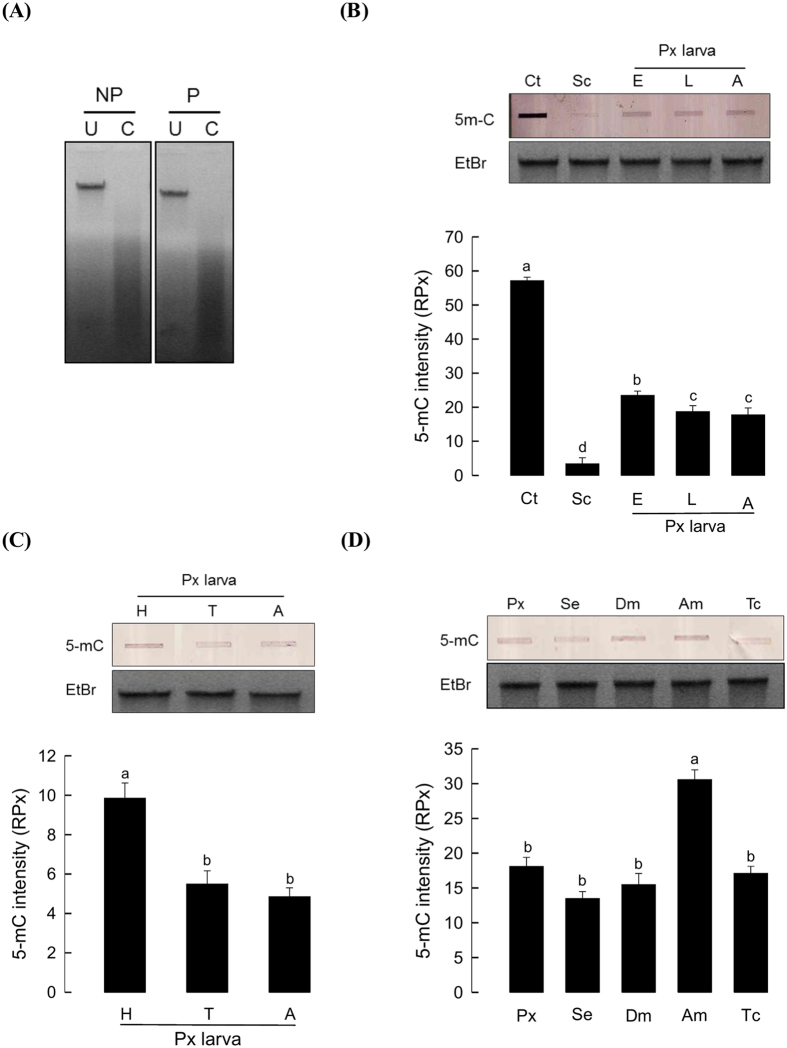
DNA methylation in *P. xylostella* (‘Px’) genome. (**A**) Restriction enzyme analysis for the presence of DNA methylation in *P. xylostella. Gla* I (4 units) digestion of *P. xylostella* genomic DNA (2.0 μg) from non-parasitized (‘NP’) or parasitized (‘P’) larvae. ‘U’ indicates undigested and ‘C’ indicates digested genomic DNAs (gDNAs) separated on 1.5% agarose gel. (**B**) Quantitative analysis of 5-methyl cytosine (5-mC) of different developmental stages of *P. xylostella:* egg (‘E’), larvae (‘L’), adult (‘A’) along with calf thymus gDNA (‘Ct’) as positive control and *Saccharomyces cerevisiae* gDNA (‘Sc’) as negative control. (**C**) Variation in DNA methylation of different tagmata of Px larvae: head (‘H’), thorax (‘T’), and abdomen (‘A’). (**D**) Comparison of Px gDNA methylation with those of different species: *Spodoptera exigua* (‘Se’), *Drosophila melanogaster* (‘Dm’), *Apis mellifera* (‘Am’), and *Tribolium castaneum* (‘Tc’). DNA methylation was detected in slot blot experiment using specific monoclonal antibody against 5-mC. Each slot contained 2.0 μg of gDNA. The experiment was repeated 3 times using different genomic DNA samples. Methylation was quantified by ImageJ analysis. ‘RPx’ represents relative pixel units on the basis of 250 ng of calf thymus gDNA (=10 RPx). EtBr represents ethidium bromide staining. Different letters above standard deviations indicate significant difference among means at Type I error = 0.05 (LSD test).

To test any presence of 5-methylcytosine (5-mC) on *P. xylostella* genome, its genomic DNA (gDNA) was digested with *Gla* I restriction enzyme to specifically cleave at 5-mC (Fig. 1A). *Gla* I cut gDNAs of both NP and P larvae of *P. xylostella*. A monoclonal antibody specific to 5-mC was used to measure the relative amounts of 5-mC on *P. xylostella* genomes (Fig. 1B). As expected, a vertebrate gDNA (a positive control) obtained from calf thymus possessed high amount of 5-mC, while yeast gDNA (a negative control) did not possess any 5-mC. The presence of 5-mC was found in different developmental stages of *P. xylostella*. The 5-mC levels were expressed as relative amounts compared to 5-mC level of the amount of calf thymus DNA (250 ng = 10 RPx, Fig. S1). There were significant differences in 5-mC levels among developmental stages. Egg had significantly (*P* < 0.05) more 5-mC level than other developmental stages. Among different body parts of larvae, the head region had higher (*P* < 0.05) levels of 5-mC than other body parts (Fig. 1C). The DNA methylation amounts of *P. xylostella* larvae were also compared among different insect species. The amount of 5-mC in *P. xylostella* gDNA was similar to that in other insects except honey bee which had the highest (*P* < 0.05) amount of DNA methylation (Fig. 1D).

### Down-regulation of 5-mC level in *P. xylostella* gDNA by *C. plutellae* parasitism

**Figure 2:**
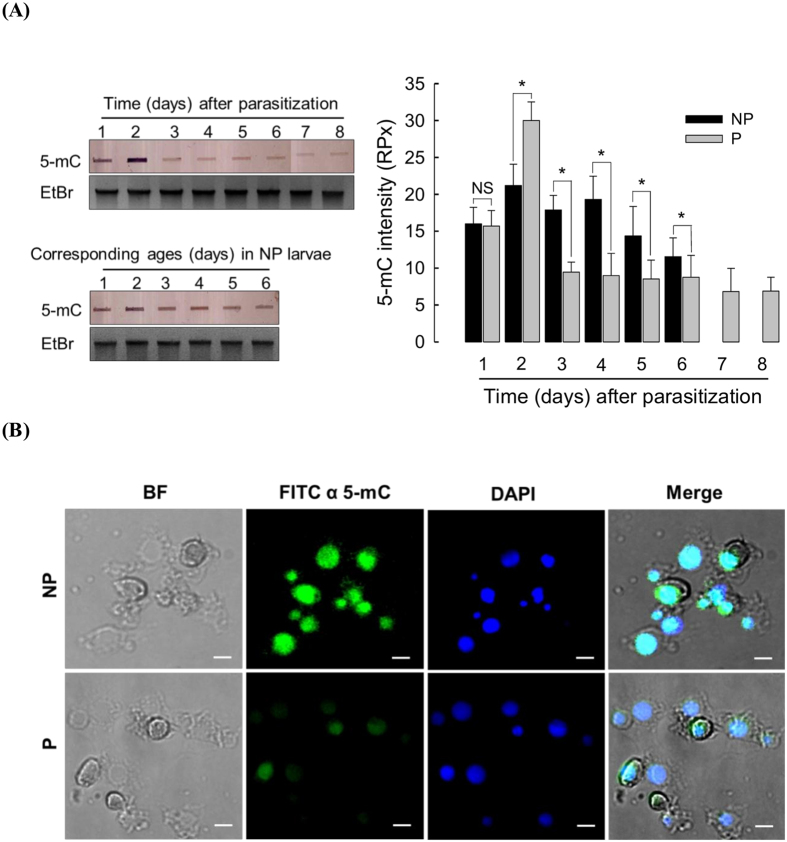
Decrease in DNA methylation of *P. xylostella* larvae parasitized by *C. plutellae*. (**A**) Decrease of 5-methyl cytosine (5-mC) in parasitized (‘P’) larvae compared to non-parasitized (‘NP’) larvae. Slot blot analysis of 5-mC (left). Each slot contains 2 μg of gDNA. Ethidium bromide (‘EtBr’) staining indicates that equal amounts of gDNA are used in different samples. Quantification of 5-mC amounts on the slot blot (right). The amounts are expressed in relative pixel (‘RPx’) units on the basis of 250 ng of calf thymus gDNA (=10 RPx). ‘NS’ and ‘*’ indicate ‘not significant difference’ or ‘significant difference’ between NP and P larvae at Type I error = 0.05 (LSD test). Each measurement was replicated three times with different biological samples. (**B**) Immunofluorescence assay of 5-mC in hemocytes of *P. xylostella* larvae. Total hemocytes were visualized at bright field (‘BF’) mode. 5-mC was detected with a monoclonal antibody and visualized with a FITC-conjugate secondary antibody. Nuclei were visualized by DAPI. Merged view combined BF, FITC, and DAPI pictures. Scale bar indicates 10 μM.

Change in 5-mC levels was monitored during the development of P larvae to determine whether parasitism could influence host DNA methylation (Fig. 2). In P larvae, 5-mC level was transiently increased at 2 days after parasitization, but significantly decreased thereafter compared to the levels in NP larvae (Fig. 2A). In contrast, NP larvae did not show any difference during the larval stages corresponding to P larvae. 5-mC was observed in the nuclei of hemocytes of NP *P. xylostella* larvae (Fig. 2B). However, its intensity was clearly decreased in the hemocytes of P larvae. P larvae contained endoparasitoid eggs or larvae within the body. Parasitoid wasp eggs and larvae also possessed 5-mC in their genomes (Fig. S2). Parasitoid wasps also showed significant variations in 5-mC levels among developmental stages, in which the egg stage had the highest (*P* < 0.05) level. Thus, 5-mC amounts assessed in P larvae represented methylation amounts in both host and parasite.

### Two different DNA methyltransferases *(DNMT-1 and DNMT-2)* of *P. xylostella*

**Figure 3:**
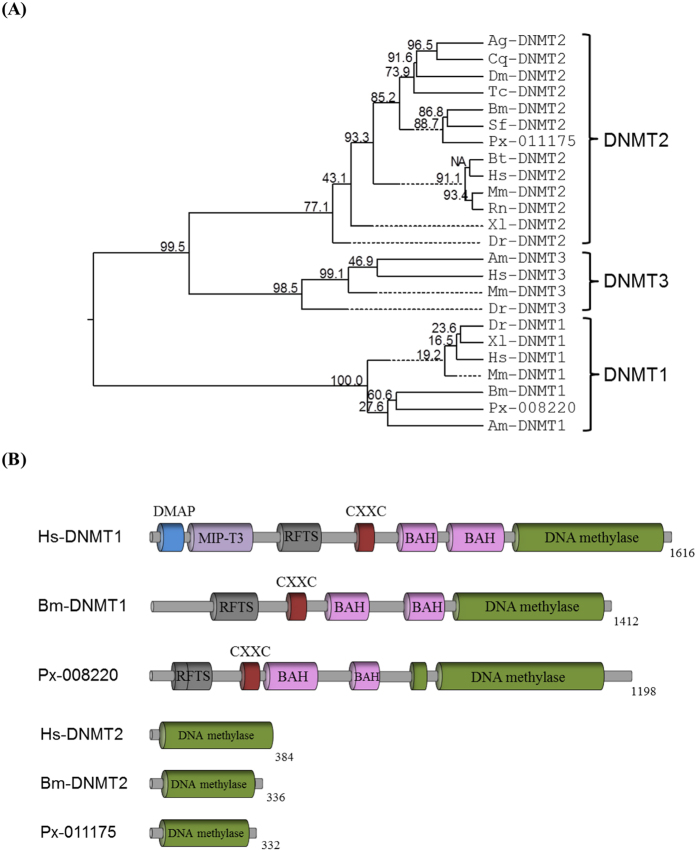
Two DNA methyltransferases *PxDNMT1* and *PxDNMT2* of *P. xylostella.* (**A**) Phylogenetic analysis of *PxDNMT1* (Px008220) and *PxDNMT2* (Px011175) with known *DNMT* genes using MEGA6^52^ and ClustalW programs of DNASTAR (Version 5.01). Bootstrap values were obtained after 500 repetitions. Amino acid sequences of *DNMT*s were retrieved from GenBank with accession numbers list in Table S1. (**B**) Schematic diagram of domain structure of *PxDNMT1* and *PxDNMT2* with reference to *Homo sapiens* (Hs) and *Bombyx mori* (Bm) *DNMT*s. Conserved domains include DNA methyltransferase 1 associated protein (DMAP), microtubule-interacting protein associated with TRAF3 (MIP-T3), replication foci targeting sequence (RFTS), Zn-finger-like motif (CXXC), bromo-associated homology (BAH), and C-5 cytosine specific DNA methylase (DNA methylase) domain predicted with Pfam domain search engine (http://pfam.xfam.org/search).

From the genome database of *P. xylostella*, two *DNMT* genes (Px008220 and Px011175) were annotated. When these two *DNMT* genes were aligned with known *DNMT* genes, Px008220 and Px011175 were clustered with *DNMT*-1 and *DNMT*-2, respectively (Fig. 3A). Domain analysis indicated that Px008220, similar to human DNMT-1, possessed DNA methyltransferase 1-associated protein (DMAP), microtubule-interacting protein associated with TRAF3 (MIP-T3), replication foci targeting sequence (RFTS), zinc finger domain (CXXC), bromo-associated homology (BAH), and 5-mC-specific DNA methylase (DNA methylase) domain (Fig. 3B, Fig. S3). However, Px011175 contained only 5-mC-specific DNA methylase (DNA methylase) domain, similar to human *DNMT*-2. These indicate that *P. xylostella* possesses two *DNMT*s: *PxDNMT1* and *PxDNMT2*.

**Figure 4:**
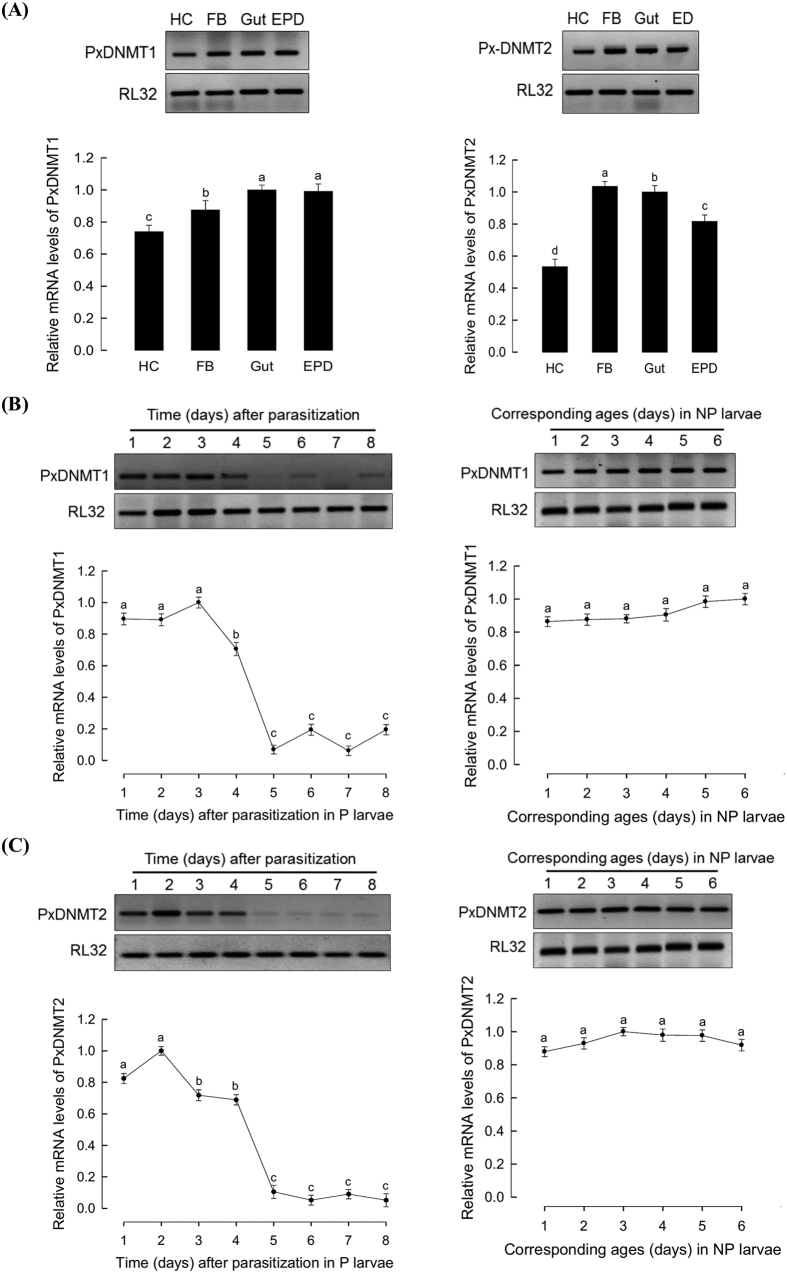
Down-regulation of *PxDNMT1* and *PxDNMT2* expressions in *P. xylostella* larvae parasitized by *C. plutellae*. (**A**) Expression profile of *PxDNMT1 and PxDNMT2* in different tissues (hemocyte (‘HC’), fat body (‘FB’), midgut (‘gut’), and epidermis (‘EPD’)) of fourth instar larvae of *P. xylostella* determined by RT-PCR (above) and RT-qPCR (below). (**B**) Decrease of *PxDNMT1* expression levels *in P. xylostella* larvae parasitized by *C. plutellae. PxDNMT1* mRNA levels in parasitized (‘P’) and nonparasitized (‘NP’) larvae determined by RT-PCR (above) and RT-qPCR (below). (**C**) Decrease of *PxDNMT2* expression levels in *P. xylostella* larvae parasitized by *C. plutellae* determined by RT-PCR (above) and RT-qPCR (below). Expression of ribosomal protein RL32 was used to confirm cDNA integrity. Each treatment was replicated three times. Different letters above standard deviations indicate significant difference among means at Type I error = 0.05 (LSD test).

Expressions of *PxDNMT1* and *PxDNMT2* were monitored in different tissues of late larvae of *P. xylostella* (Fig. 4A). All tested tissues expressed these two *DNMTs*. However, the expression patterns were different between the two *DNMT*s. *PxDNMT1* in the gut or epidermis was expressed more than that in the hemocyte or fat body. In contrast, fat body exhibited the highest expression level of *PxDNMT2*. The expression levels of two *DNMT*s in NP and P larvae were compared (Fig. 4B). In NP larvae, the expression levels of the two *DNMT*s were not changed as larvae grew. However, in P larvae, the expression levels of both *DNMT*s were significantly (*P* < 0.05) decreased as larvae grew. The expression level of *PxDNMT1* was significantly (*P* < 0.05) decreased at 4 days post parasitization, while that of *PxDNMT2* was significantly (*P* < 0.05) decreased at 3 days post parasitization (Fig. 4C).

### Influence of parasitism on expression of *MBD* genes of *P. xylostella*

**Figure 5:**
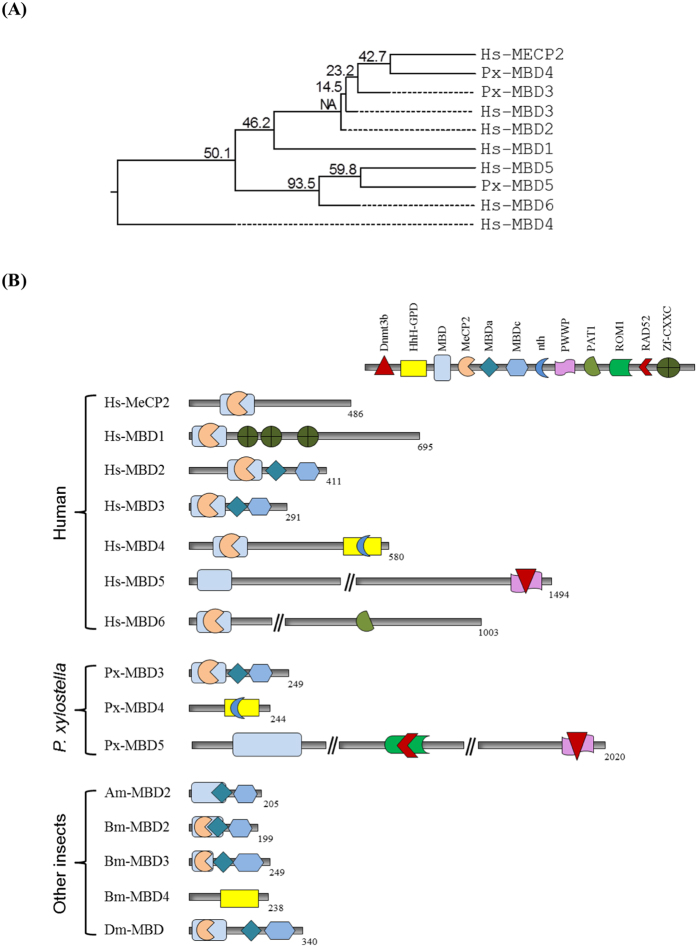
Three methylation-binding domain (*MBD*) proteins of *P. xylostella*. (**A**) Phylogenetic analysis of *PxMBD3* (Px003962), *PxMBD4* (XP_011549067.1), and *PxMBD5* (Px004348) compared to other types of human *MBD*s using MEGA6^52^ and ClustalW programs of DNASTAR (Version 5.01). Bootstrap values were obtained after 500 repetitions. Amino acid sequences of human *MBD*s were retrieved from GenBank with accession numbers listed in Table S1. (**B**) Schematic diagram of domain structure of *PxMBD*s with reference to human and other insect species. Conserved domains include DNA methyltransferase 3b (Dnmt3b), hallmark helix-hairpin-helix and Gly/Pro rich loop domain (HhH-GPD), methyl-CpG binding domain (MBD), methyl-CpG-binding protein 2 (MeCP2), methyl-CpG binding domain at P55 region (MBDa), methyl-CpG binding protein C-terminal (MBDc), endonuclease III (nth), Pro-Trp-Trp-Pro (PWWP), DNA topoisomerase II-associated protein PAT1 (PAT1), retinal outer segment membrane protein 1 (ROM1), DNA recombination/repair protein Rad52 (RAD52), and zinc finger CXXC domain (Zf-CXXC). They were predicted with Pfam domain search engine (http://pfam.xfam.org/search).

5-mC can be recognized by specific recognition protein *MBD* that recruits and mediates chromatin remodeling to control gene expression^23^. Three *MBD* genes (*PxMBD3, PxMBD4*, and *PxMBD*5) were annotated in the DBM genome database (Fig. 5). These three *PxMBD*s share high sequence homologies with human *MBD*s (Fig. 5A). Especially, *PxMBD4* was co-clustered with a canonical MBD protein, MeCP2, of human. In domain analysis, all three *PxMBD*s possess the characteristic MBD domain (Fig. 5B, Fig. S4).

**Figure 6:**
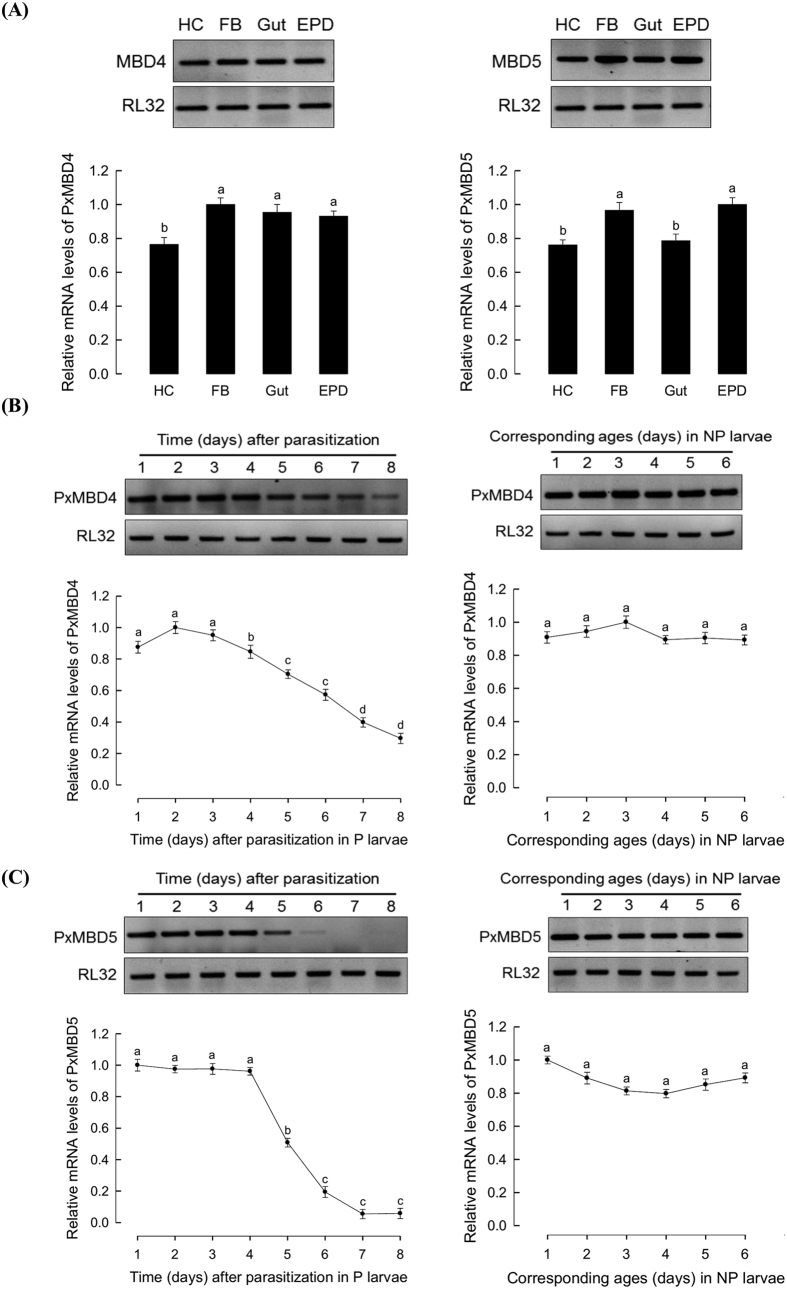
Expression patterns of three *MBD* genes of *P. xylostella.* (**A**) Expression levels of *PxMBD4* and *PxMBD5* in different tissues (hemocyte (‘HC’), fat body (‘FB’), midgut (‘gut’), and epidermis (‘EPD’)) of fourth instar larvae of *P. xylostella* determined by RT-PCR (above) and RT-qPCR (below). (**B**) Changes in *PxMBD4* expression levels in *P. xylostella* larvae parasitized by *C. plutellae. PxMBD4* mRNA levels in parasitized (‘P’) or nonparasitized (‘NP’) larvae were determined by RT-PCR (above) and RT-qPCR (below). (**C**) Decrease of *PxMBD5* expression levels in *P. xylostella* larvae parasitized by *C. plutellae* based on RT-PCR (above) and RT-qPCR (below). Expression of ribosomal protein RL32 was used to confirm cDNA integrity. Each treatment was replicated three times. Different letters above standard deviations indicate significant difference among means at Type I error = 0.05 (LSD test).

Out of the three *MBD*s of *P. xylostella*, only *PxMBD4* and *PxMBD5* were analyzed for their expression levels in NP and P larvae of *P. xylostella* (Fig. 6) because the expression of *PxMBD3* was not detected in our RT-PCR assay. All tested tissues expressed these two *MBD*s, in which fat body and epidermis exhibited higher expression levels of the two *MBD*s than hemocyte or gut (Fig. 6A). The expression levels of the two *MBD*s in NP and P larvae were compared (Fig. 6B). In NP larvae, the expression levels of the two *MBD*s were not changed during development. However, in P larvae, the expression levels of both *MBD*s were significantly (*P* < 0.05) decreased as larvae grew. Expression level of *PxMBD4* in NP larvae was significantly (*P* < 0.05) decreased at 4 days post parasitization, while that of *PxMBD5* was significantly (*P* < 0.05) decreased at 5 days post parasitization (Fig. 6C).

### Influence of parasitism on DNA methylation-degrading enzyme *(TET)* of *P. xylostella*

**Figure 7:**
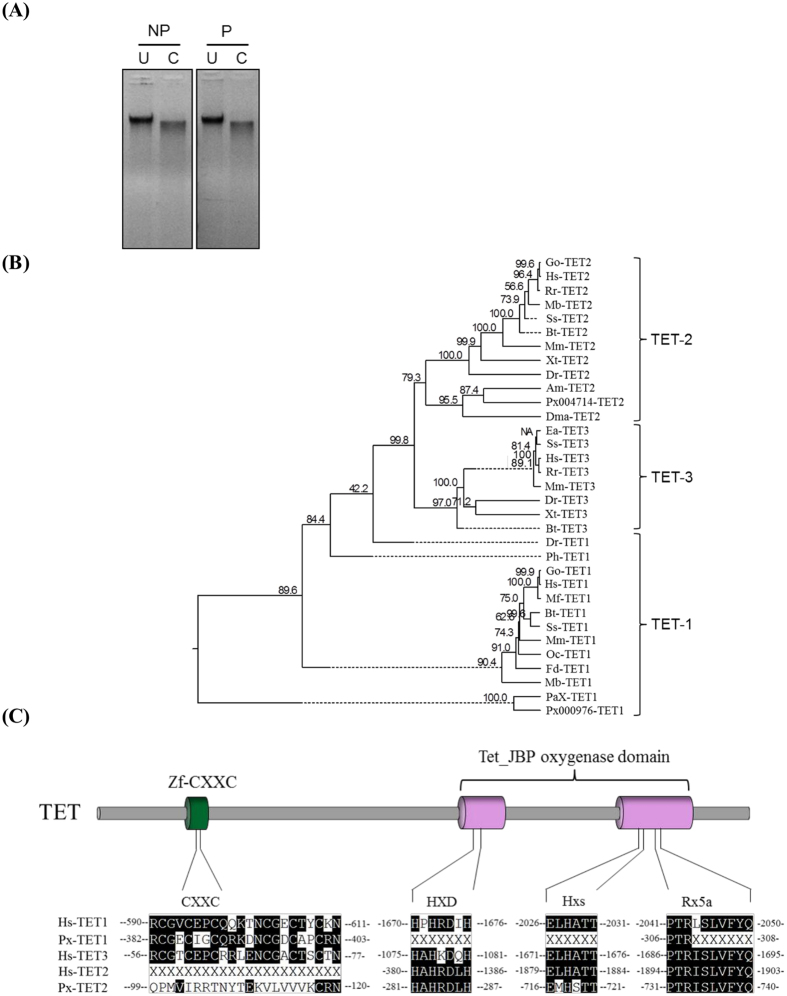
DNA methylation-degradation enzyme (*ten-eleven translation (PxTET*)) in *P. xylostella*. (**A**) Restriction enzyme analysis for the presence of DNA hydroxymethylation in *P. xylostella. PvuRts*1I (1 unit) digestion of *P. xylostella* genomic DNA (2.0 μg) from nonparasitized (‘NP’) and parasitized (‘P’) larvae. ‘U’ indicates undigested and ‘C’ indicates digested genomic DNA (gDNAs) separated on 1.5% agarose gel. (**B**) Phylogenetic analysis of *PxTET1* (Px000976) and *PxTET2* (Px004714) with known TET genes using MEGA6^52^ and ClustalW programs of DNASTAR (Version 5.01). Bootstrap values were obtained after 500 repetitions. Amino acid sequences of TETs were retrieved from GenBank with accession numbers listed in Table S1. (**C**) Schematic diagram of domain structure of *PxTET1* and *PxTET2* with reference to human *TET*s. Conserved domains include zinc finger CXXC (Zf-CXXC) and 2-oxoglutarate and iron-dependent dioxygenase (Tet_JBP) containing HxD, Hxs, and Rx5a motifs. All domains and motifs were predicted with Pfam domain search engine (http://pfam.xfam.org/search).

Methylation levels on cytosine residues were modulated earlier than the down-regulation of *DNMT* expression, suggesting that there might be an expressional control in methylation-degrading enzyme before down-regulation of *DNMT* expression after parasitization. *PvuRts*1I is an enzyme that specifically digests 5-hydroxymethylcytosine. Indeed, this restriction enzyme could digest both NP and P larvae genomic DNAs, supporting the presence of DNA methylation-degrading enzyme (Fig. 7A). From the *P. xylostella* genome database, Px000976 (*PxTET1*) and Px004714 (*PxTET2*) were annotated to be TET dioxygenase, a DNA methylation-degrading enzyme (Fig. 7B). Based on their predicted amino acid sequences, both *PxTET1* and *PxTET2* shared similar structures with human TET. They all possessed two characteristic domains of zinc finger CXXC (Zf-CXXC) and Tet_JBP (2-oxoglutarate and iron-dependent dioxygenase) containing HxD, Hxs, and Rx5a motifs (Fig. 7C).

**Figure 8:**
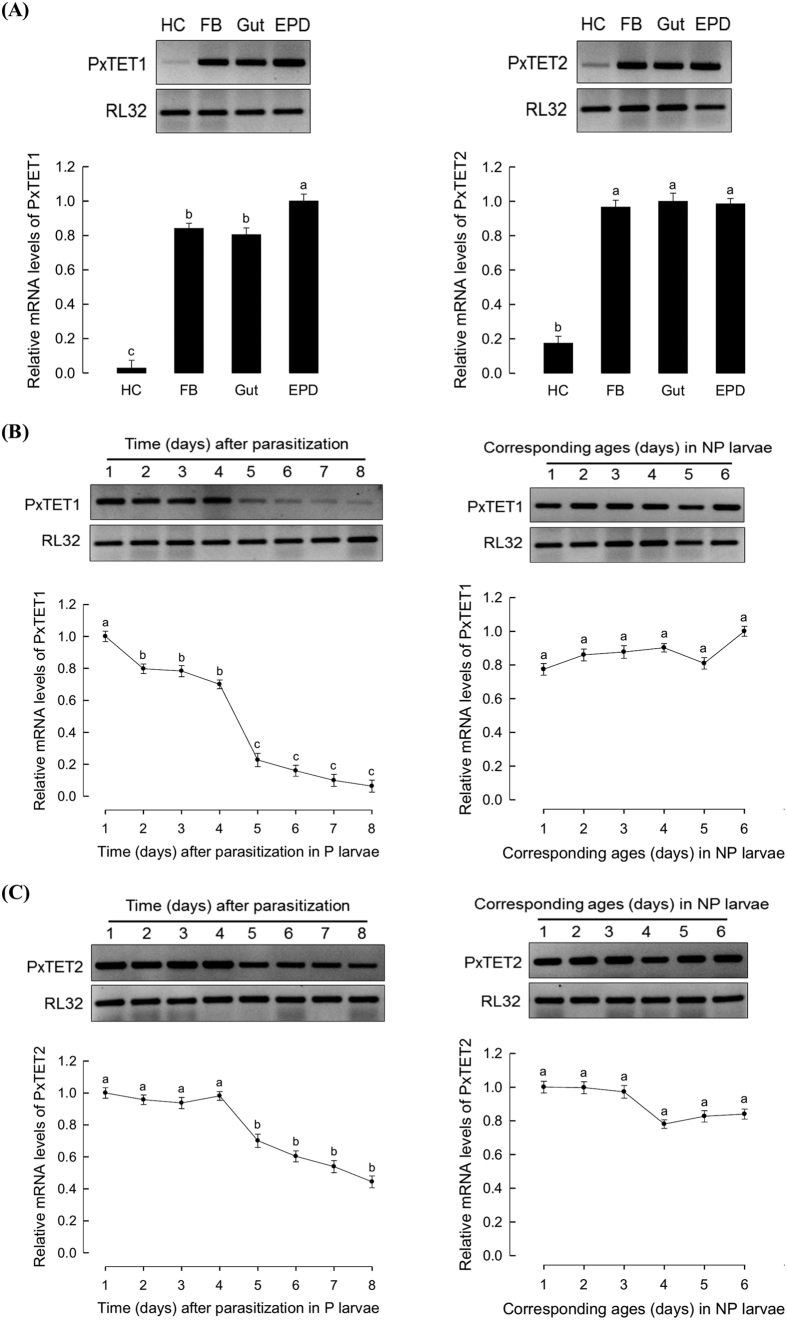
Expression patterns of *ten-eleven translation proteins (TET*s) in *P. xylostella.* (**A**) Expression profile of *PxTET1 and PxTET2* in different tissues (hemocyte (‘HC’), fat body (‘FB’), mid gut (‘gut’), and epidermis (‘EPD’)) of fourth instar larvae of *P. xylostella* based on RT-PCR (above) and RT-qPCR (below). (**B**) Down-regulation of *PxTET1* in *P. xylostella* larvae parasitized by *C. plutellae. PxTET1* mRNA levels in parasitized (‘P’) and nonparasitized (‘NP’) larvae based on RT-PCR (above) and RT-qPCR (below) are shown. (**C**) Down-regulation of *PxTET2* in *P. xylostella* larvae parasitized by *C. plutellae* based on RT-PCR (above) and RT-qPCR (below). Expression of a ribosomal protein RL32 was used to confirm cDNA integrity. Each treatment was replicated three times. Different letters above standard deviations indicate significant difference among means at Type I error = 0.05 (LSD test).

The expression levels of both *PxTET*s were assessed in different tissues of *P. xylostella* larvae. Results are shown in Fig. 8A. All tissues exhibited marked expressions of the two *TET*s, in which hemocytes exhibited the lowest expression levels of both *TET*s. The expression levels of the two TETs in NP and P larvae were compared (Fig. 8B). In NP larvae, the expression levels of both *TET*s were not changed during larvae development. However, in P larvae, the expression levels of both *TET*s were significantly decreased as larvae grew. Expression level of *PxTET1* was significantly (*P* < 0.05) decreased at 2 days post parasitization, while that of *PxTET2* was significantly (*P* < 0.05) decreased at 5 days post parasitization (Fig. 8C).

### Significance of 5-mC in *P. xylostella* genome in immune response and development

**Figure 9:**
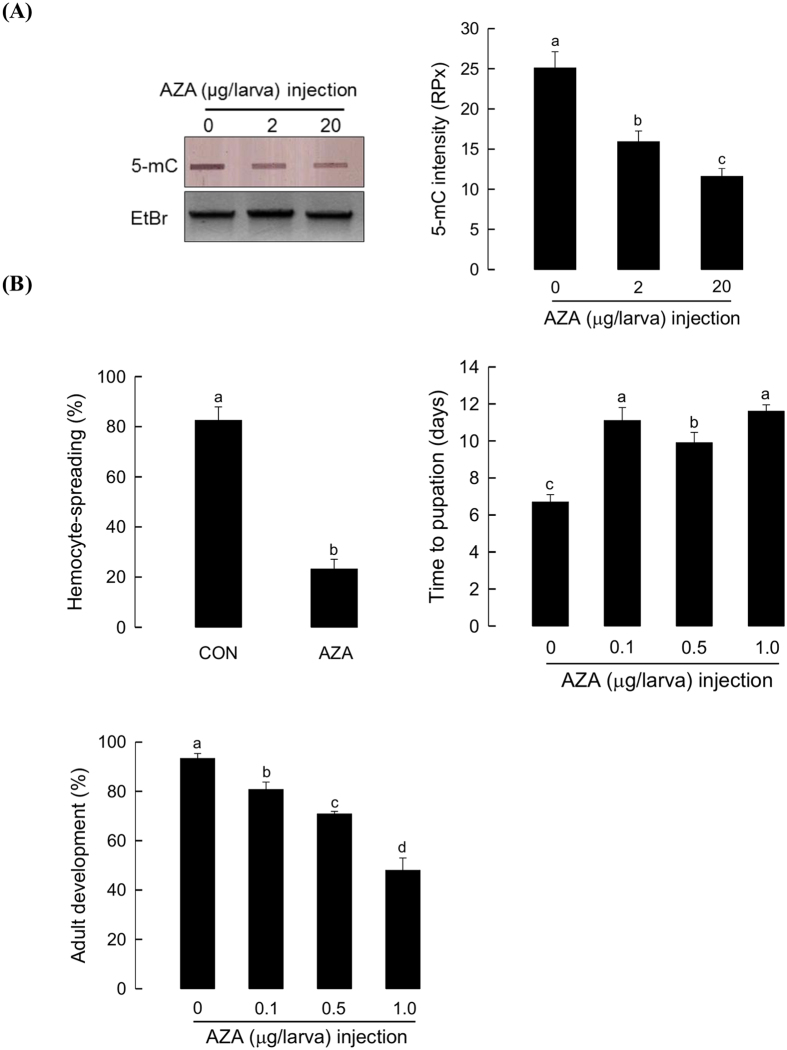
Influence of 5-Aza-2′-deoxycytidine (AZA) on immune response and development of *P. xylostella*. (**A**) Dose-dependent inhibitory effect of AZA on 5-methyl cytosine (5-mC) levels. Its inhibitory effect was assessed at different concentrations (0, 2.0, and 20 μg/larva) of AZA at 48 h post-injection to third instar larvae. For slot blot of 5-mC (left), each slot contained 2.0 μg genomic DNA (gDNA). Quantification of 5-mC amounts of the slot blot bands is shown in the right panel. The amounts are expressed in relative pixel (RPx) units on the basis of 250 ng of calf thymus gDNA (=10 RPx). Ethidium bromide stain indicates that equal amounts of gDNA are used in different samples. As control, solvent DMSO was injected. Each treatment was replicated three times. (**B**) Effect of AZA on immune response and development of *P. xylostella.* For measurement of immune response, hemocyte-spreading behavior was checked at 48 h after the injection of 2 μg of AZA. Hemocyte-spreading behavior was assessed by counting cytoplasmic extension along with filopodial or pseudopodial growth. As control, DMSO was injected. Each treatment used 10 larvae. For measurement of developmental alterations, AZA (2 μg) was injected to each first day third instar (L3D1) larvae. For pupation time (days), time to pupation was measured from L3D1 to pupation. Adult emergence (%) was observed after complete escape from pupal cuticle. Thirty individuals were assessed in each treatment. Different letters above standard deviations indicate significant difference among means at Type I error = 0.05 (LSD test).

AZA, a specific inhibitor of cytosine methylation, was used to test the significance of 5-mC in immune response and developmental processes of *P. xylostella*. AZA treatment at 48 h significantly (*P* < 0.05) decreased 5-mC levels of *P. xylostella* gDNA in a dose-dependent manner (Fig. 9A). In addition, AZA treatment significantly (*P* < 0.05) suppressed immune response based on hemocyte-spreading behavior (Fig. 9B). The AZA treatment also delayed the development of *P. xylostella* from larvae to pupae.

**Figure 10:**
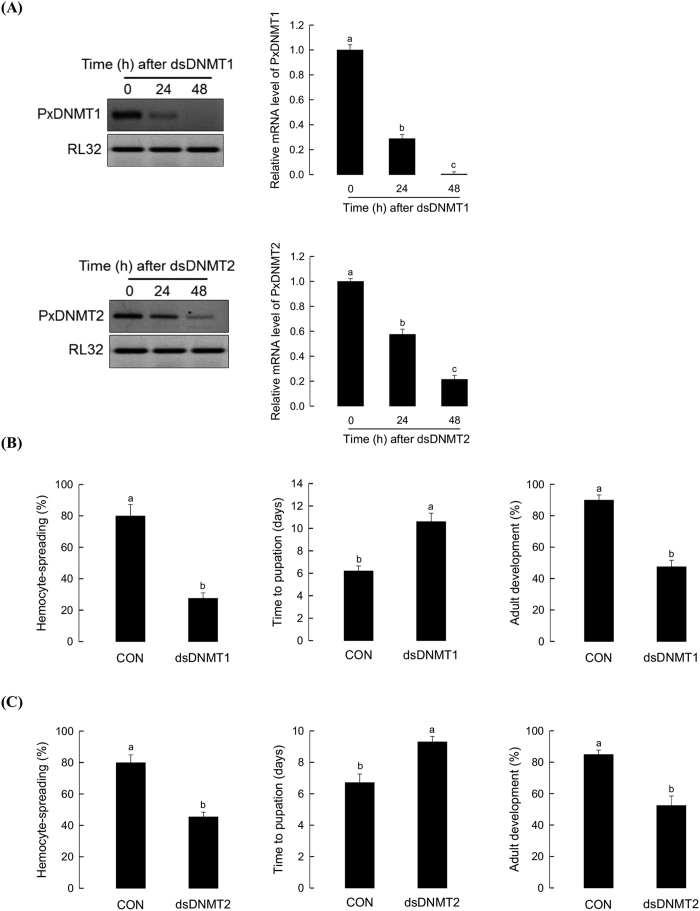
Influence of RNA interference (RNAi) of *PxDNMT1* and *PxDNMT2* on immune response and development of *P. xylostella*. (**A**) RNAi efficacy of *PxDNMT*s. After injection of dsRNAs specific to *PxDNMT1* (dsDNMT1) or *PxDNMT2* (dsDNMT2) (250 ng dsRNA per larva), RNAi effect was assessed after 24 and 48 h of dsRNA injection by RT-PCR (left) and RT-qPCR (right). RT-qPCR was replicated three times. (**B,C**) Effect of RNAi of *PxDNMT1* or *PxDNMT2* on immune response and development of *P. xylostella.* For measurement of immune response, hemocyte-spreading behavior was checked at 48 h post injection of 250 ng of dsDNMT1 or dsDNMT2. Hemocyte-spreading was assessed by counting cytoplasmic extension along with filopodial or pseudopodial growth. As control dsRNA (dsCON), dsRNA specific to a viral gene CpBV-ORF302 was injected. Each treatment used 10 larvae. For measurement of developmental alterations, dsDNMT1 or dsDNMT2 was injected to each first day third instar (L3D1) larvae. For pupation time (days), time to pupation was measured from L3D1 to pupation. Adult emergence (%) was observed after complete escape from pupal cuticle. Thirty individuals were assessed in each treatment. Different letters above standard deviations indicate significant difference among means at Type I error = 0.05 (LSD test).

To confirm the results of AZA assay, both *PxDNMT1* and *DNMT2* expressions were suppressed by RNA interference (Fig. 10). Treatment with dsRNAs specific *PxDNMT1* and *PxDNMT2* significantly (*P* < 0.05) suppressed the expression of *PxDNMT1* and *PxDNMT2* expression, respectively, in the NP larvae of *P. xylostella* (Fig. 10A). Larvae treated with dsRNA specific to *PxDNMT1* (Fig. 10B) or *PxDNMT2* (Fig. 10C) exhibited immune response and development similar to larvae treated with AZA.

## Discussion

DNA methylation is a way to transmit epigenetic information to the next generation. It is a covalent modification of DNA by adding methyl group mostly to cytosine at CpG sequence in animals with catalytic activities of DNMTs. Once methylated, the methylated sites can be recognized by 5-methyl-CpG-binding domain proteins (*MBD*s), which will recruit chromatin remodeling machineries to control gene expression^24^. DNA methylation can be changed by various environmental factors as described in Introduction section. This study demonstrated that a novel environmental factor could change DNA methylation with respect to parasite and host interaction, in which an endoparasitoid wasp, *C. plutellae*, suppressed *DNMT* expressions in order to alter host DNA methylation status to induce immunosuppression and delay developmental rate of host that are necessary for the development of immature endoparasitoid wasp.

Whole-genome methylation analyses in insects such as honeybee (*Apis mellifera*), silkworm moth (*Bombyx mori*), and parasitic wasp (*Nasonia vitripennis*) have shown that 5-mC is by far the most common form of programmed DNA modification^25^,^26^,^27^. This current study showed that *P. xylostella* genomic DNA was methylated. Two methods can be used to observe the DNA methylation of *P. xylostella* genome. A restriction enzyme, *Gla* I, can be used to digests XCGX with methylated cytosine^28^. Both NP and P genomic DNAs were susceptible to *Gla* I treatment. Slot blot assay using a monoclonal antibody specifically recognizing 5-mC showed positive reactions to both NP and P larval gDNAs. Densitometry analysis after slot blot assay indicated that the relative amounts of 5-mC on *P. xylostella* gDNAs were similar among different developmental stages, including eggs, larvae, and adults. It also showed that DNA methylation level of *P. xylostella* was much lower than that of a mammalian species, but was relatively similar among insects such as coleopteran, dipteran, and lepidopteran species except hymenopteran specie. About 4–5% of the cytosines are usually methylated on the entire chromosomes of vertebrates^29^, while only 0.5% or less of cytosine residues are methylated in *Drosophila* spp.^19^. This suggests that *P. xylostella* genome has methylated cytosines at around 0.5% among total cytosines. Most invertebrates have relatively low DNA methylation amounts in their genome. However, they exhibit the persistence of DNA methylation^30^,^31^. In contrast to genome-wide DNA methylation in vertebrates, DNA methylation in invertebrates is largely confined to genes and most of them are not in the intergenic regions^32^,^33^. Especially, DNA methylation on transposable and repetitive elements is almost nonexistent in insects^34^,^35^. These facts suggest that DNA methylation plays little role in suppressing the proliferation of transposable elements in insects, unlike vertebrates. Indeed, intragenic DNA methylation characteristic of insects is involved in gene expression through mRNA splicing^36^. Clearly, DNA methylation plays crucial roles in insect physiological processes. Gene-specific DNA methylation can mediate insecticide resistance by up-regulating esterase gene expression to increase detoxification in the green peach aphid, *Myzus persicae*^37^. In the European honey bee, *A. mellifera*, DNA methylation controls alternative splicing of mRNAs in the fat body^38^. These results suggest that any alteration of DNA methylation may cause significant physiological alterations in *P. xylostella*.

Parasitism by *C. plutellae* suppressed *DNMT* gene expressions of *P. xylostella*. Two *DNMT* genes, *PxDNMT1* and *PxDNMT2*, were identified from *P. xylostella* genome. PxDNMT-1 is likely to play a role in maintaining DNA methylation status because it shares structural similarity with human DNMT-1. In contrast, PxDNMT2 is highly homologous to human DNMT-2. Thus, *P. xylostella* has two types of DNMT. However, it does not have DNMT-3. In insects, there are variations in retaining *DNMT* genes in their genome^18^. Hemipteran and hymenopteran species possess all three types of *DNMT* genes. In the pea aphid, *Acyrthosiphon pisum*, two isoforms of DNMT1 and DNMT3 are encoded in its genome^39^. In addition, honey bee and other social hymenopteran insects possess both DNMTs that presumably perform maintenance of DNA methylation (DNMT-1) and *de novo* DNA methylation (DNMT-3)^40^,^41^. However, coleopteran and lepidopteran species possess only DNMT-1 and DNMT-2, but not DNMT-3^31^. Furthermore, dipteran species do not have DNMT-1 or DNMT-3. They only have DNMT-2^19^,^42^. Thus, lepidopteran species including *P. xylostella* analyzed in this study appear to lose DNMT-3, indicating that lepidopteran species may not form *de novo* DNA methylation during development in their life cycle. However, epigenetic control through DNA methylation plays a significant role in domestication of silkworm from a wild population, in which demethylation is associated with down-regulation of gene expression during domestication^26^. Furthermore, DNMT-1 of *B. mori* has been confirmed to have DNA methylation activity and its knockdown of gene expression will lead to decreased egg hatch^26^,^43^. This suggests that there must be *de novo* DNA methylation in *B. mori*. A structural prediction of DNMT-2 suggests that it may perform DNA methylation in cytosines of non-CpG sequences^44^. Thus, the two DNMTs encoded in *P. xylostella* may play roles in maintaining DNA methylation and *de novo* DNA methylation to epigenetically control gene expression. Thus, suppression of both *PxDNMT1* and *PxDNMT2* in P larvae would significantly alter the physiological processes of *P. xylostella*. In fact, down-regulation of *DNMT* gene expression resulted in decreased 5-mC contents in *P. xylostella*. Significant decrease of 5-mC levels in P larvae began at 3 days after parasitization. This appears to be well explained by significant decrease of *PxDNMT2* expression in P larvae. However, there was a significant increase of 5-mC level in P larvae at 2 days after parasitization. This may be explained by changes in 5-mC levels in parasitoid egg or larvae within host P larvae. Assessment of 5-mC levels in different *C. plutellae* developmental stages showed that 5-mC levels were decreased with development (Fig. S2). Although P larvae at 2 days after parasitization had egg or larvae of parasitoid wasp, P larvae at 1 day after parasitization also had the parasitoid eggs that had the highest 5-mC level. Thus, the increase of 5-mC level in P larvae at 2 days after parasitization is not likely to be explained by the 5-mC of endoparasoids. Alternatively, such increase might be due to decreased degradation of DNA methylation by host metabolizing enzyme such as *TET* that can demethylate 5-mC by oxidation processes^45^. The expressional levels of both *TET* genes were decreased during parasitism. Interestingly, the expression level of *PxTET1*, but not *PxTET2*, began to decrease at 2 days after the parasitization. This suggests that the early increase of 5-mC level in the P larvae might be due to decreased 5-mC metabolism by *TET* under no change in expression levels of both *DNMT*s. However, any causal relationship between down-regulation of *PxTET1* expression and increase of 5-mC level is yet clear until an activity of PxTET1 to catalyze demethylation of 5-mC is confirmed. The transient increase may be significant in terms of host defense because high methylation on host genome is not favored for parasitoid development (see below). Decrease of 5-mC levels is associated with physiological alterations such as immunosuppression and delay of larval development. AZA is a specific inhibitor of 5-mC in mammals^46^. AZA treatment significantly decreased 5-mC level in *P. xylostella* and altered its physiological processes. Similarly, RNAi of *PxDNMT1* or *PxDNMT2* resulted in significant physiological alterations. These results suggest that DNA methylation is required for normal physiological processes of *P. xylostella*. In addition, endoparasitoid wasp *C. plutellae* can alter host physiological processes through an epigenetic mode by suppressing *DNMT* expressions. DNA methylation is closely associated with insect development for cell growth and differentiation^47^. The best example is the royal jelly effect on queen development of *A. mellifera*, in which royal jelly contains a specific inhibitor to histone deacetylase (HDAC) that is linked to DNMT3^48^. The HDAC inhibitor can reduce DNA methylation level to extend larval period. With respect to immunity, epigenetic control plays a crucial role in modulating gene expression of antimicrobial peptides (AMPs). It has been reported that histone acetylation can promote AMP expression in the greater wax moth, *Galleria mellonella*^49^. In *Drosophila*, DNMT-2 is required for efficient innate immune responses especially in antiviral defense by directly binding to viral RNAs with subsequent modification through RNA methylation^50^. Considering that *C. plutellae* depends on its symbiotic virus CpBV for successful parasitism, the parasitoid wasp should suppress *PxDNMT2*. The question is, which parasitic factor suppresses the expression of *PxDNMT*s? As explained in the Introduction, several CpBV genes can modulate host gene expression. Among these CpBV genes, vH4 might modulate host gene expressions by chromatin remodeling through direct joining to host nucleosomes^9^. This prediction needs to be explored in subsequent studies. Alternatively, down-regulation of DNA methylation in parasitized larvae of *P. xylostella* may be indirect due to alteration of host gene expression by various parasitic factors as demonstrated in another parasitoid wasp against *P. xylostella*^4^.

In summary, *P. xylostella* genome is methylated and its methylation levels can be modulated by its methylation and demethylation machineries. Environmental factor such as parasitism by *C. plutellae* can influence its host genome by altering DNA methylation through significant reduction in gene expression of both *DNMT-1* and *DNMT-2*. The reduction of host DNA methylation might be accompanied by massive changes in gene expression. This epigenetic alteration of host gene expression would favor successful parasitism of *C. plutellae*.

## Methods

### Insects and parasitization

All procedures are described in Park and Kim^51^.

### Bioinformatics for DNA methylation-associated genes

From a genome database of *P. xylostella* (www.iae.fafu.edu.cn/DBM) and NCBI-GenBank, the following genes were obtained: two *DNA methyltransferase* genes (*DNMT-1*, Px008220; *DNMT-2*, Px011175), three *methyl-binding domain* proteins (*MBD-3*, Px003962; *MBD-4*, XP_011549067.1; and *MBD-5*, Px004348), and two *ten-eleven translation* proteins (*TET-1*, Px000976; *TET-2*, Px004714). Predicted amino acid sequences of these *P. xylostella* genes were then compared to those of other genes obtained from GenBank (Table S1).

Phylogenetic analyses were performed using MEGA6^52^ and ClustalW programs of DNASTAR (Version 5.01, Madison, WI, USA). Bootstrapping values to support branching were obtained with 500 repetitions. Protein domains of these genes were predicted using Pfam version 29.0 (http://pfam.xfam.org) provided by European Bioinformatics Institute.

### RNA interference (RNAi)

Double-stranded RNAs (dsRNAs) were prepared using a method described by Park and Kim^51^. For microinjection of dsRNA, each dsRNA was mixed with a cell transfection reagent (Metafectene PRO, Biontex, Planegg, Germany) in a ratio of 1:1 (v/v) and incubated at 25 °C for 20 min. As dsRNA control, dsRNA specific to a viral gene *CpBV-ORF302*^51^ was used. A hundred nL of dsRNA (250 ng) complex was injected into each hemocoel of early third instar larvae of *P. xylostella* and its effect on gene expression was quantified by RT-qPCR as described below.

### Real time-quantitative PCR (RT-qPCR)

Total RNA extraction and cDNA preparation followed a method described in Park and Kim^51^. RT-qPCR was performed in 20 μL reaction volume consisting of 2× SYBR^®^ Green Realtime PCR Master Mix (TOYOBO, Dojima Hama, Osaka, Japan), 5 μM of gene-specific forward and reverse primers (Table S2), and 50 ng of cDNA as template. As an endogenous control, *RL32* gene was assessed along with test samples. PCR was performed at 95 °C for 5 min for initial denaturation followed by 40 cycles at 98 °C for 15 s, 54 °C for 30 s, and 72 °C for 45 s with a final extension at 72 °C for 7 min. Melting curves were assessed to confirm unique PCR products. Relative quantification method^53^ was used to estimate mRNA expression levels. The experiment was independently replicated three times.

### Analysis of DNA methylation using slot-blot apparatus

gDNAs were isolated from different developmental stages of *P. xylostella* larvae. DNA concentration and purity were determined with GeneQuant spectrophotometer and electrophoresis on 1.5% agarose gel, respectively. Two μg of gDNA was first denatured by heating at 100 °C for 10 min in a tube containing denaturing solution (0.4 M NaOH, 10 mM EDTA). The reaction was neutralized by adding equal volume of 2 mM ice cold ammonium acetate (pH 7.0). As positive and negative controls of DNA methylation, a calf thymus DNA (Sigma-Aldrich Korea, Seoul, Korea) and *Saccharomyces cerevisiae* yeast DNA (Invitrogen, Seoul, Korea) were used, respectively. After denatured DNA samples were then passed through slot-blot apparatus (Bio Rad, Hercules, CA, USA), the membranes were air-dried and exposed twice to a UV crosslinker (CL-1000, UVP, LLC, Upland, CA, USA) for each 30 sec. After blocking the membranes with 5% skimmed milk, membranes were incubated with primary antibody raised against 5-mC (Abcam, Seoul, Korea) at 1:1,000 dilution for 2 h. The bound primary antibody was captured with secondary antibody conjugated with alkaline phosphatase (Sigma-Aldrich Korea) and visualized with 5-bromo-4-chloro-3-indolyl phosphate/nitro blue tetrazolium (Sigma-Aldrich Korea). For densitometry quantification of bands, the membranes were air-dried and images were captured with HP Scanjet 5470c scanner (Hewlett-Packard Company, Greeley, CO, USA). Band intensity was estimated using ImageJ 2.1.4.9 software (http://www.freewarefiles.com/downloads_counter.php?programid=53729). At every run, 250 ng of calf thymus DNA was loaded to normalize band intensity and calculate relative pixel (RPx) amounts, in which 25 ng of calf thymus DNA was set to be 1 RPx.

### Restriction enzyme assay

Purified gDNA was digested with 4 units of *Gla* I (SibEnzyme, West Roxbury, MA, USA) at 30 °C for 16 h or 1 unit of *PvuRts*1I (Diagenode, Seraing, Belgium) at 32 °C for 2 h in a total volume of 50 μL. The resulting DNA was separated on 1.5% agarose gel and stained with ethidium bromide.

### Analysis of DNA methylation using immunofluorescence assay

For fluorescence microscope observation, hemocytes were collected from 50 larvae of *P. xylostella* in anticoagulant buffer (ACB: 1.32% sodium citrate, 0.48% citric acid, and 1.47% dextrose, pH 4.5). The suspension was incubated on ice for 40 min and replaced with TC-100 insect cell culture medium (Hyclone, Daegu, Korea). The hemocyte suspension was transferred onto glass slide and incubated at 25 °C for 12 h in the dark. After washing once with 100 mM phosphate buffered saline (PBS, pH 7.4), cells were fixed with 4% paraformaldehyde at RT for 10 min and permeabilized with cold methanol (−20 °C) for 2 min. Cells were washed with PBS and incubated with primary antibody against 5-mC (1:5,000 dilution) at RT for 1 h. After washing twice in PBS, secondary antibody-conjugated with fluorescein isothiocyanate (FITC, Sigma-Aldrich Korea) was added in 1:5,000 dilutions and incubated at RT for 1 h. Cells were washed twice in PBS and incubated with 4′,6-diamindino-2-phenylindole (DAPI, Thermo Scientific, Meridian, Rockford, USA) at 1:1,000 dilution in PBS for 2 min. After washing twice with PBS, cells were observed under a fluorescence microscope (DM 2500, Leica, Wetzlar, Germany) at 400× magnification.

### 5-Aza-2′-deoxycytidine (AZA) treatment

AZA was purchased from Sigma-Aldrich Korea and dissolved in dimethylsulfoxide (DMSO, Amresco, Solon, Ohio, USA) to prepare 50 mg/mL stock solution. For microinjection, AZA was mixed with Metafectene PRO as described above. DMSO was used as control. At 24–48 h post injection, total gDNA was isolated from larvae to assess methylation levels using slot blot analysis. Other larvae treated with AZA were subjected to assessment of hemocyte-spreading behavior assay or developmental assessment (see below).

### Hemocyte-spreading behavior assay

Hemolymph was collected in ACB from NP larvae of *P. xylostella* at 48 h after injection of AZA or dsRNA specific to *PxDNMT1* or *PxDNMT2*. After centrifuging at 800× *g* for 3 min, the pellet was resuspended in equal volume of ice cold ACB solution and incubated on ice for 20 min. The suspension was centrifuged and the pellet was resuspended in 1 mL of TC100 insect cell culture medium. Fifty μL of hemocyte suspension was seeded onto 96 well culture plates and incubated at 25 °C for 40 min in the dark. Hemocyte-spreading behavior was observed under a phase contrast microscope (Olympus S730, Tokyo, Japan) by counting hemocytes exhibiting cytoplasmic extension such as filopodial growth and pseudopodial growth.

### Developmental assay

NP larvae at L3D1 stage were microinjected with 0.1, 0.5, and 1.0 μg of AZA and 250 ng of dsRNA specific to *PxDNMT1* or *PxDNMT2* as described above. Treated larvae were fed with cabbage leaves *ad libitum* and kept at 25 °C for growth. For pupal development assessment, time to pupation was measured by the elapsed time from L3D1 to pupation. Pupation was confirmed by pupal molting within cocoon. For adult emergence assessment, whole body escape from the pupal cuticle was considered as complete adult emergence. The number of adult emerged was counted. All bioassays used three replicates per treatment. For each replication, 30 larvae were used.

## Additional Information

**How to cite this article**: Kumar, S. and Kim, Y. An endoparasitoid wasp influences host DNA methylation. *Sci. Rep.* **7**, 43287; doi: 10.1038/srep43287 (2017).

**Publisher's note:** Springer Nature remains neutral with regard to jurisdictional claims in published maps and institutional affiliations.

## Supplementary Material

Supplementary Data
