# Supplementary material for: An endoparasitoid wasp influences host DNA methylation
Source: Sci Rep. 2017 Feb 23;7:43287. doi: 10.1038/srep43287 (PMC5322367; doi:10.1038/srep43287)
Supplement: Supplementary Data [file srep43287-s1.pdf]

Scientific Reports

Supplementary data

An endoparasitoid wasp influences host DNA methylation

Sunil Kumar & Yonggyun Kim*

Department of Bioresource Sciences, Andong National University, Andong 36729, Korea

* Correspondence and requests for materials should be addressed to Y.K. (email: hosanna@anu.ac.kr)

Supplementary data

Table S1. List of NCBI-GenBank or DBM genome database (www.iae.fafu.edu.cn/DBM) and accession numbers of genes used in phylogenetic analyses

Table S2. List of primers used in this study

Figure S1. Quantification of 5-mC in relative pixels of calf thymus DNA on slot blot. Calf thymus (positive control) genomic DNA (gDNA) was 2-fold serially diluted and transferred onto nitrocellulose membrane and detected with specific monoclonal antibody against 5-mC. Ethidium bromide stain indicates respective 2-fold serially diluted amount of gDNA. Band intensity was assessed with ImageJ program. The experiment was replicated three times.

Figure S2. Quantitative analysis of 5-methyl cytosine (5-mC) of different developmental stages of *Cotesia plutellae:* egg ('E'), larva ('L'), pupa ('P'), and adult ('A') of nonparasitized ('NP') larvae compared to parasitized ('P') *P. xylostella* larvae. Ethidium bromide stain indicates equal amounts of genomic DNA (gDNA) of different samples are used. DNA methylation was detected in slot blot experiment using specific monoclonal antibody against 5-mC. Each slot contained 2.0 μg of gDNA. The experiment was replicated three times. Methylation was quantified by ImageJ analysis. 'RPx' represents relative pixel units on the basis of 250 ng of calf thymus gDNA (= 10 RPx). Different letters above standard deviations indicate significant difference among means at Type I error = 0.05 (LSD test).

Figure S3. Phylogenetic analysis of *PxDNMT1* and *PxDNMT2* in *P. xylostella*. (A, B) Protein based phylogenetic analysis were obtained with MEGA6[51] and ClustalW program of DNASTAR (Version 5.01) showing homology among different *DNMT1* and *DNMT2* genes. Bootstrapping values were obtained with 500 repetitions. Amino acid sequences of DNMTs were retrieved from GenBank with accession numbers listed in Table S1.

Figure S4. Phylogenetic analysis of *methyl-binding domain* (MBDs) in *P. xylostella*. Protein based phylogenetic analysis was obtained using MEGA6[51] and ClustalW program of DNASTAR (Version 5.01) to show homologies among different MBD genes. Bootstrapping values were obtained with 500 repetitions. Amino acid sequences of *DNMT*s were retrieved from GenBank with accession numbers listed in Table S1.

Table S1. List of NCBI-GenBank or DBM genome database (www.iae.fafu.edu.cn/DBM) and accession numbers of genes used in phylogenetic analyses

Group	Genes	Species	Accession numbers
DNMT-1	*AmDNMT*-1	*Apis mellifera*	XP_006562865
	BmDNMT-1	*Bombyx mori*	LC010238
	DrDNMT-1	*Danio rerio*	AAI63894
	HsDNMT-1	*Homo sapiens*	P26358
	MmDNMT-1	*Mus musculus*	P13864
	PxDNMT-1	*Plutella xylostella*	Px008220
	XlDNMT-1	*Xenopus laevis*	AAH72774
DNMT-2	*AgDNMT*-2	*Anopheles gambiae*	XP_312975.5
	BmDNMT-2	*Bombyx mori*	NP_001036934.1
	BtDNMT-2	*Bos taurus*	NP_861528.1
	CqDNMT-2	*Culex quinquefasciatus*	XP_001867327.1
	DmDNMT-2	*Drosophila melanogaster*	NP_001036355.1
	DrDNMT-2	*Danio rerio*	NP_001018153.1
	HsDNMT-2	*Homo sapiens*	AAC39764.1
	MmDNMT-2	*Mus musculus*	AAC53529.1
	PxDNMT-2	*Plutella xylostella*	Px011175
	RnDNMT-2	*Rattus norvegicus*	NP_001026813.1
	SfDNMT-2	*Spodoptera frugiperda*	AFS64716.1
	TcDNMT-2	*Tribolium castaneum*	XP_008196999.1
	XlDNMT-2	*Xenopus laevis*	AAH46854.2
DNMT-3	*AmDNMT*-3	*Apis mellifera*	ADH84015.1
	DrDNMT-3	*Danio rerio*	AAI62467.1
	HsDNMT-3	*Homo sapiens*	AAD53063.1
	MmDNMT-3	*Mus musculus*	AAH07466.1
MBDs	*AmMBD*-2	*Apis mellifera*	XP_003250633.1
	BmMBD-2	*Bombyx mori*	XP_004929676.1
	BmMBD-3	*Bombyx mori*	XP_004929675.1
	BmMBD-4	*Bombyx mori*	XP_004928908.1

	DmMBD	*Drosophila melanogaster*	NP_001262421.1
	Hs-MECP-2	*Homo sapiens*	P51608.1
	Hs-MBD-1	*Homo sapiens*	Q9UIS9.2
	Hs-MBD-2	*Homo sapiens*	Q9UBB5.1
	Hs-MBD-3	*Homo sapiens*	O95983.1
	Hs-MBD-4	*Homo sapiens*	NP_003916.1
	Hs-MBD-5	*Homo sapiens*	NP_060798.2)
	Hs-MBD-6	*Homo sapiens*	NP_443129.3
	Px-MBD-3	*Plutella xylostella*	Px003962
	Px-MBD-4	*Plutella xylostella*	XP_011549067.1
	Px-MBD-5	*Plutella xylostella*	Px004348
TET-1	*BtTET*-1	*Bos taurus*	XP_015316519.1
	DrTET-1	*Danio rerio*	AHE93329.1
	FdTET-1	*Fukomys damarensis*	XP_010606255.1
	GoTET-1	*Gorilla gorilla*	XP_004049552.1
	HsTET-1	*Homo sapiens*	NP_085128.2
	MmTET-1	*Mus musculus*	Q3URK3.2
	MfTET-1	*Macaca fascicularis*	XP_005565748.1
	MbTET-1	*Myotis brandtii*	EPQ14278.1
	OcTET-1	*Oryctolagus cuniculus*	XP_008268265.1
	PxTET-1	*Plutella xylostella*	Px000976
	PaXTET-1	*Papilio xuthus*	KPJ01611.1
	PhTET-1	*Pseudopodoces humilis*	XP_005530562.1
	SsTET-1	*Sus scrofa*	NP_001302701.1
TET-2	*AmTET*-2	*Apis mellifera*	XP_006561262.1
	BtTET-2	*Bos taurus*	XP_005198640.1
	DrTET-2	*Danio rerio*	AHE93330.1
	DmaTET-2	*Daphnia magna*	JAM74472.1
	GoTET-2	*Gorilla gorilla*	XP_004040272.1
	HsTET-2	*Homo sapiens*	NP_001120680.1
	MmTET-2	*Mus musculus*	NP_001035490.2
	MbTET-2	*Myotis brandtii*	EPQ11582.1
	PxTET-2	*Plutella xylostella*	Px004714

	RrTET-2	*Rhinopithecus roxellana*	XP_010369845.1
	SsTET-2	*Sus scrofa*	XP_003129326.3
	XtTET-2	*Xenopus tropicalis*	XP_002934823.2
TET-3	*BtTET*-3	*Bos taurus*	XP_015320803.1
	DrTET-3	*Danio rerio*	NP_001314874.1
	EaTET-3	*Equus asinus*	XP_014685289.1
	HsTET-3	*Homo sapiens*	XP_005264244.1
	MmTET-3	*Mus musculus*	XP_006505836.1
	RrTET-3	*Rhinopithecus roxellana*	XP_010380032.1
	SsTET-3	*Sus scrofa*	XP_005662507.1
	XtTET-3	*Xenopus tropicalis*	NP_001090656.1

Table S2. List of primers used in this study

Genes	Direction	Uses	Sequences (5`→ 3’)
DNMT1	Forward	RT-PCR	GACGATACCTGAGTTGGATGAC
	Reverse	RT-PCR	GCCTTAGTCCAGGCTTTCTT
	Forward	RT-qPCR	CATCATGGAGAGACCTCAAACC
	Reverse	RT-qPCR	CTCATGTCATTCTCCACCAGAC
	Forward	RNAi	TAATACGACTCACTATAGGGAGAGACGATACCTGAGTT
	Reverse	RNAi	TAATACGACTCACTATAGGGAGAGCCTTAGTCCAGGCTT
DNMT2	Forward	RT-PCR	CTGGAACTATACAGTGGCATAGG
	Reverse	RT-PCR	TGGCAAGGTGGAGACATTAG
	Forward	RT-qPCR	CACCAGTGCCTTGGAACTAT
	Reverse	RT-qPCR	AGCCTCTGGGTCTAGGATATT
	Forward	RNAi	TAATACGACTCACTATAGGGAGACTACCCAGAGCCTGA
	Reverse	RNAi	TAATACGACTCACTATAGGGAGACAGGATCTCTTGCTGT
MDB4	Forward	RT-PCR	CAGGAGCCTCTTCTTATCCAAC
	Reverse	RT-PCR	CTCTCAGCCAATCAACGTACA
	Forward	RT-qPCR	TCCTTGGGCTTTACTCATAGC
	Reverse	RT-qPCR	TCCATCGAGCAGACAGAAAC
MDB5	Forward	RT-PCR	GCTACAAGGACGACGCTAAA
	Reverse	RT-PCR	CTTCACCACTCACACCTCTATTC
	Forward	RT-qPCR	GTGTGAGTGGTGAAGGTGATAG
	Reverse	RT-qPCR	CGGACTAACATAGAGCACAGAAG
TET1	Forward	RT-PCR	CACCAGAACGGGAGCTTAAT
	Reverse	RT-PCR	TTGTCTGTTTCTCGCTGGAG
	Forward	RT-qPCR	CCAGCGAGAAACAGACAAAGA
	Reverse	RT-qPCR	TGCAGATCTGATGCGACTTG
TET2	Forward	RT-PCR	GACAGTCAAATGGGTGGAGTAG
	Reverse	RT-PCR	GGCTTCCACTAACGCTGATAA
	Forward	RT-qPCR	GTTGTTCGTGGTCGATGTACTA
	Reverse	RT-qPCR	CGTGCATTCGCTCTTCTATCT

T7	promoter	RNAi	TAATACGACTCACTATAGGGAGA
RL32	Forward	PCRqPCR	ATGCCCAACATTGGTTACGG
RL32	Reverse	PCRqPCR	TTCGTTCTCCTGGCTGCGGA

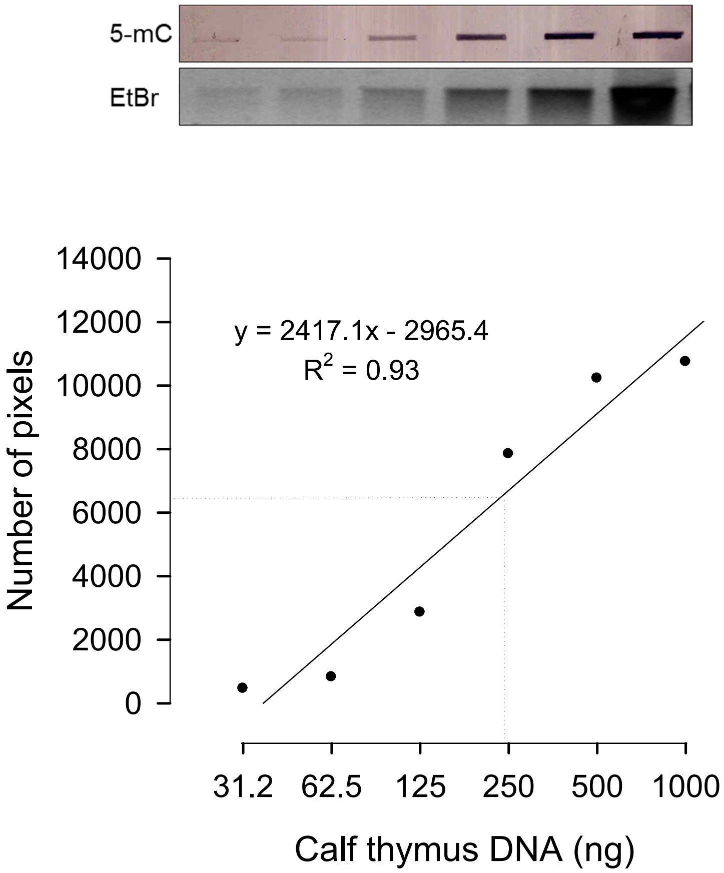
5-mC
EtBr
14000
12000
10000
8000
6000
4000
2000
0
y = 2417.1x - 2965.4
R2 = 0.93
Number of pixels
31.2
62.5
125
250
500
1000
Calf thymus DNA (ng)

Figure S1

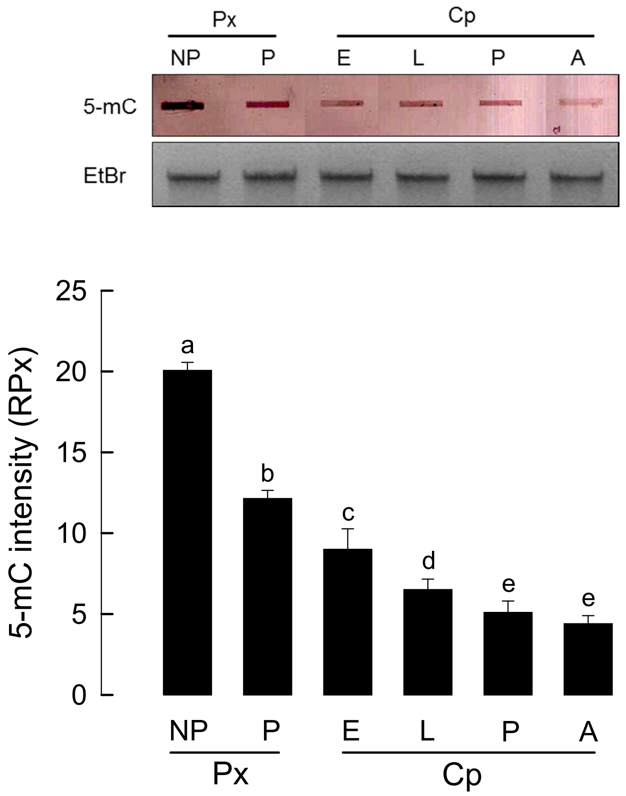

Px
Cp
NP
P
E
L
P
A
5-mC
EtBr
25
20
15
10
5
0
5-mC intensity (RPx)
a
b
c
d
e
e
NP
P
E
L
P
A
Px
Cp

Figure S2

(A)

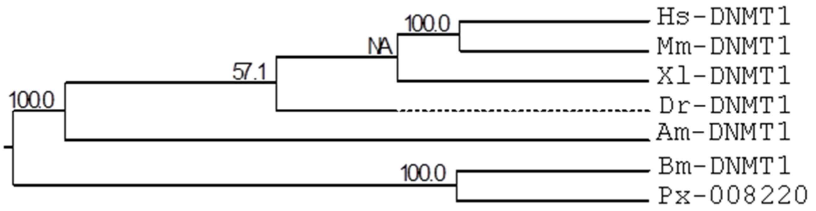

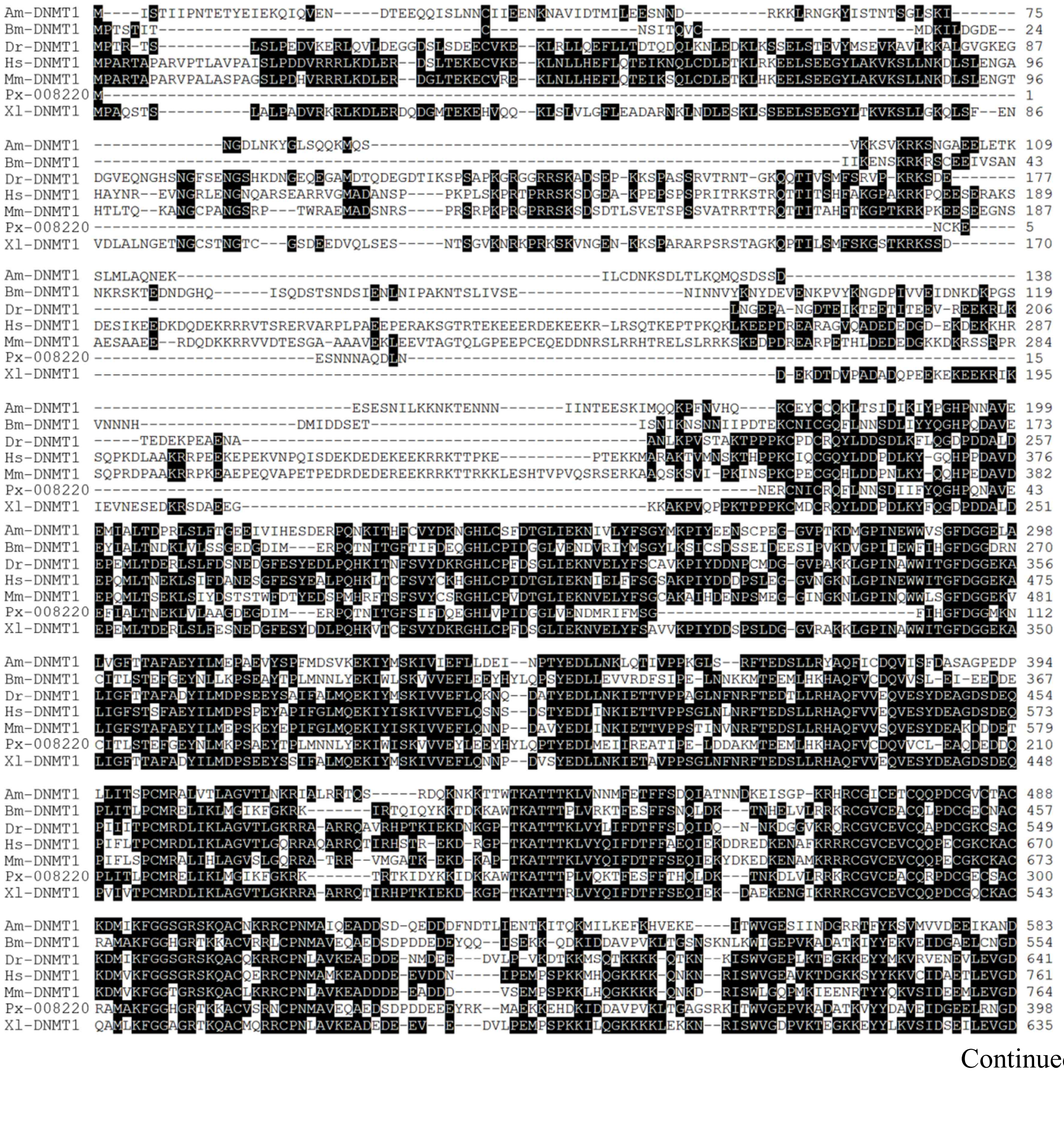

Continued...

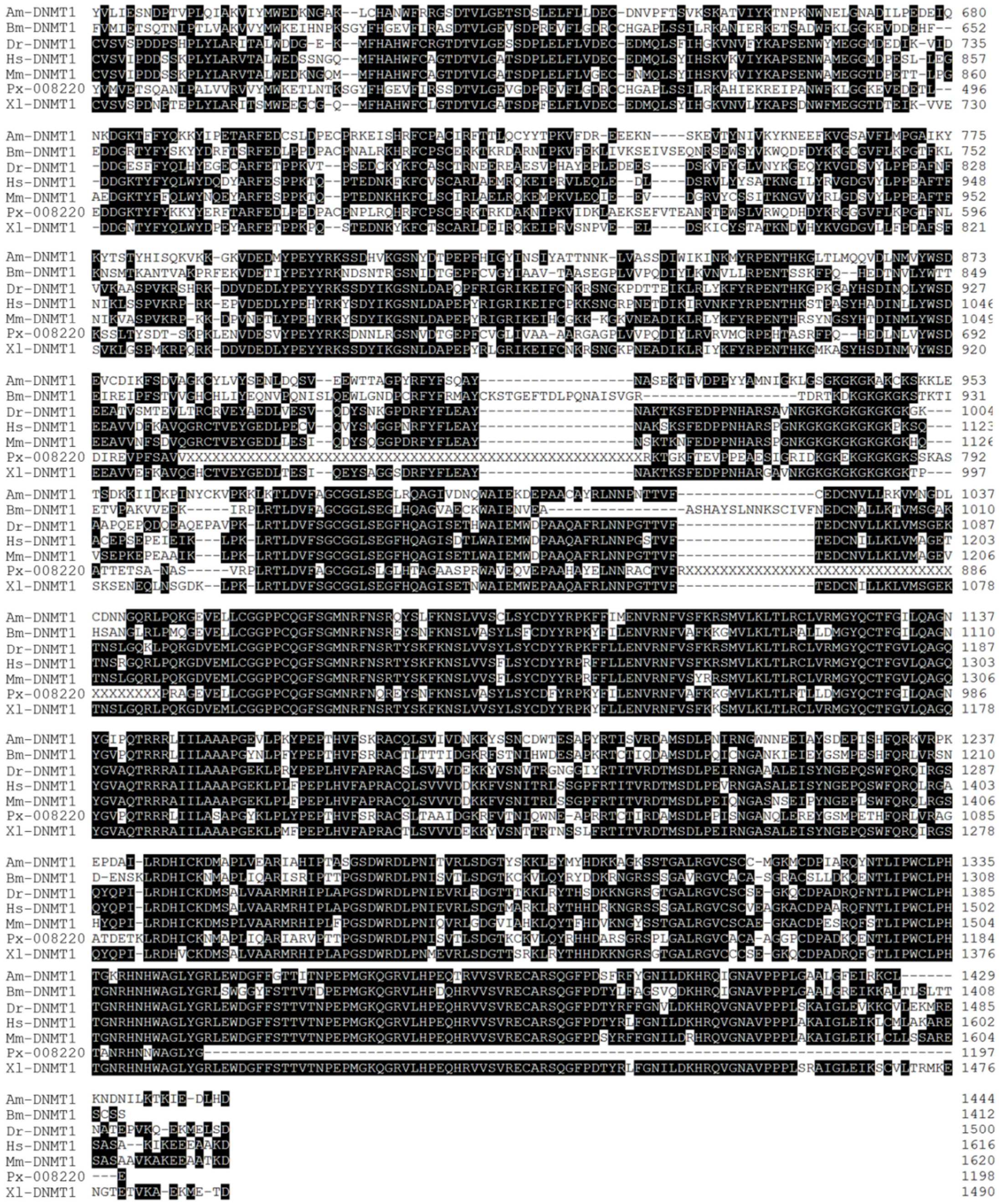

(B)

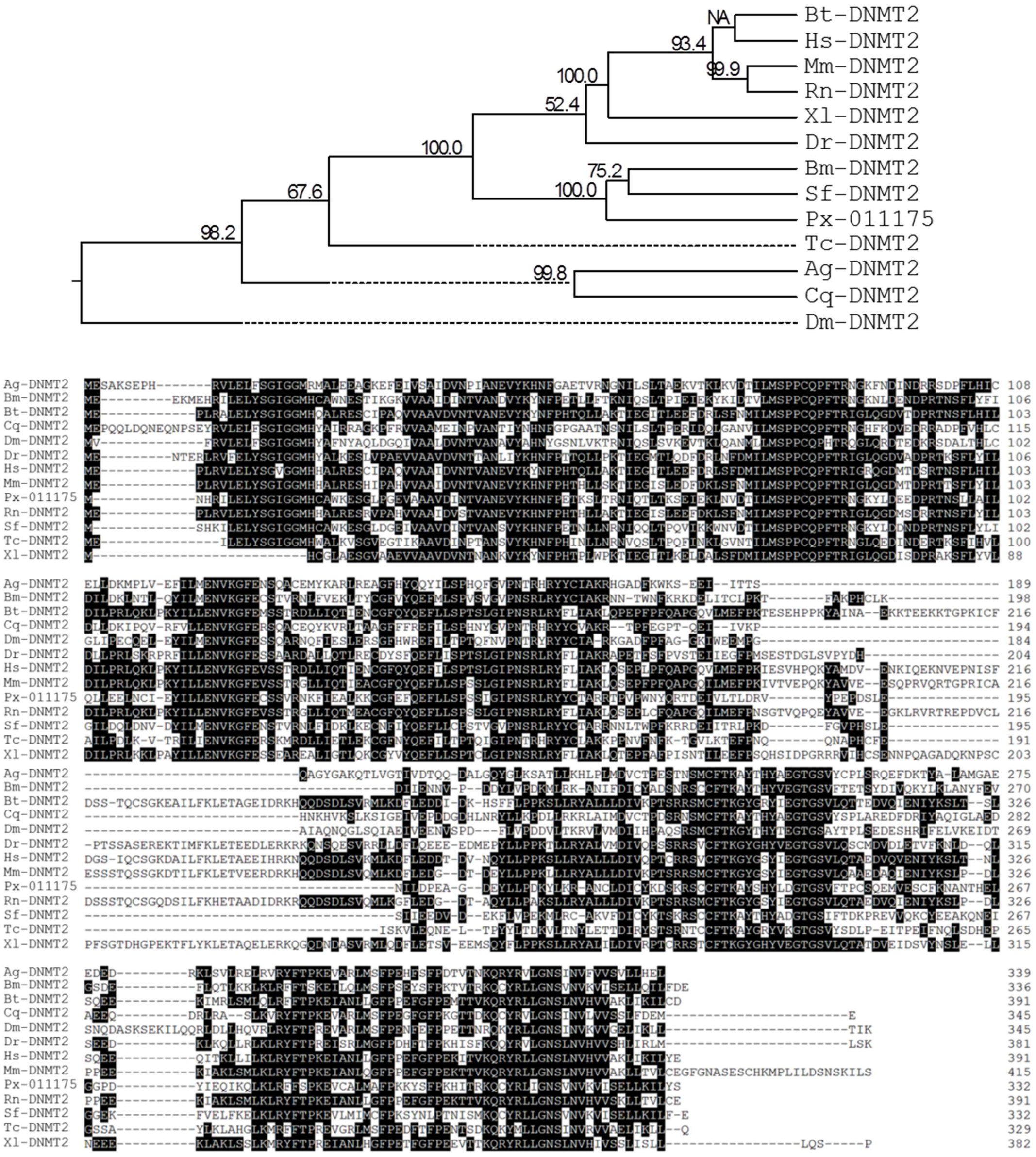

NA
93.4
99.9
100.0
52.4
100.0
75.2
100.0
67.6
98.2
99.8
Bt-DNMT2
Hs-DNMT2
Mm-DNMT2
Rn-DNMT2
Xl-DNMT2
Dr-DNMT2
Bm-DNMT2
Sf-DNMT2
Px-011175
Tc-DNMT2
Ag-DNMT2
Cq-DNMT2
Dm-DNMT2

Figure S3

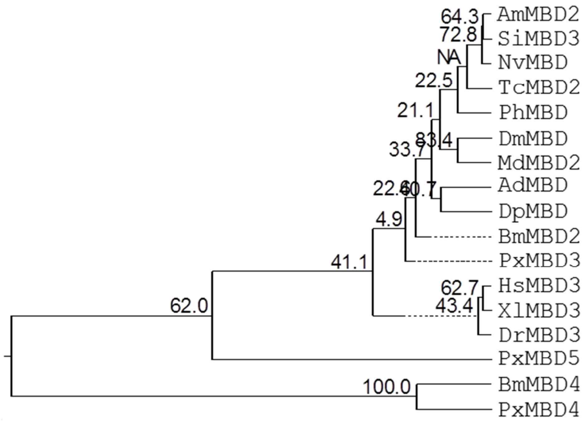
AmMBD2
SiMBD3
NvMBD
TcMBD2
PhMBD
DmMBD
MdMBD2
AdMBD
DpMBD
BmMBD2
PxMBD3
HsMBD3
XlMBD3
DrMBD3
PxMBD5
BmMBD4
PxMBD4
64.3
72.8
NA
22.5
21.1
83.4
33.7
22.6
60.7
4.9
41.1
62.7
43.4
62.0
100.0

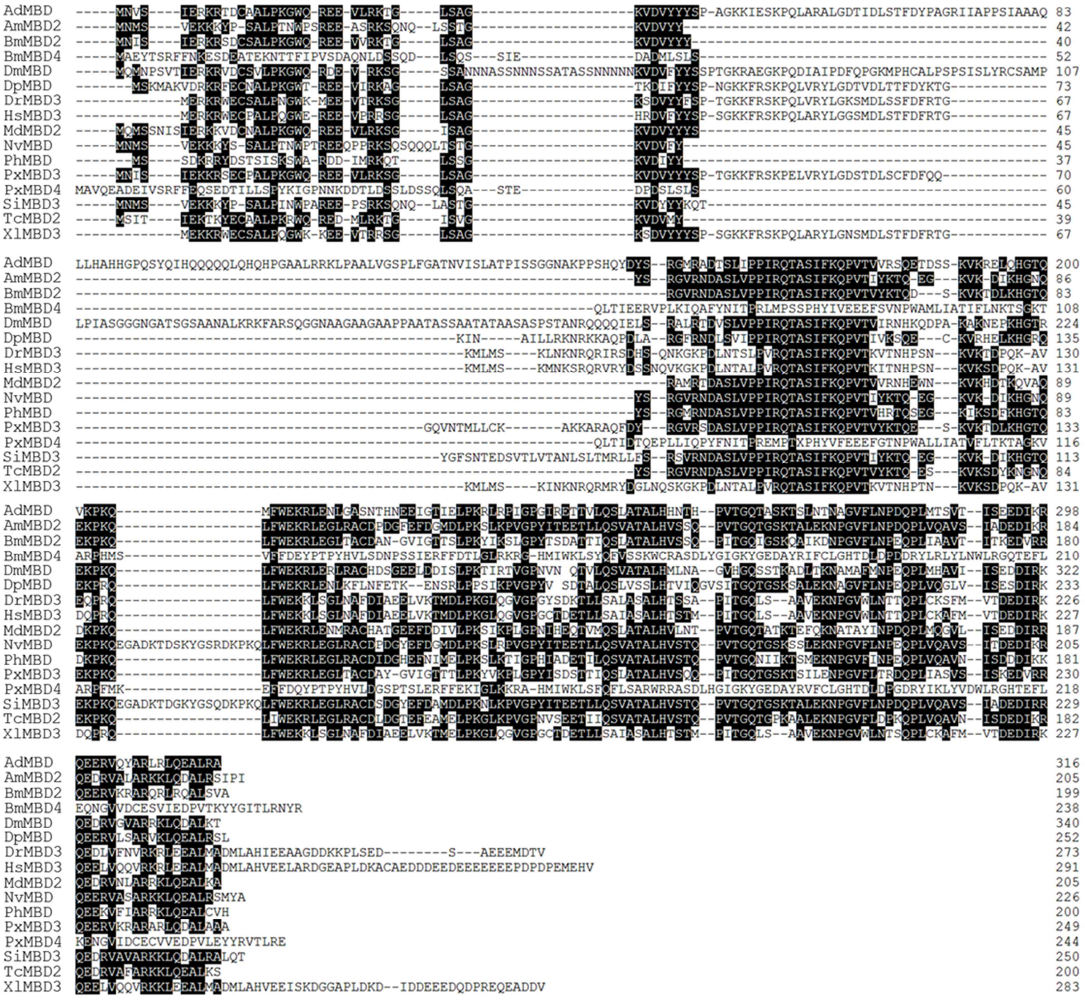

Figure S4
